# Involvement of DNA Repair Genes and System of Radiation-Induced Activation of Transposons in Formation of Transgenerational Effects

**DOI:** 10.3389/fgene.2020.596947

**Published:** 2020-11-27

**Authors:** Elena Yushkova

**Affiliations:** Department of Radioecology, Institute of Biology of Komi Scientific Centre of the Ural Branch of the Russian Academy of Science, Syktyvkar, Russia

**Keywords:** γ-rays, DNA damage, transposons, transgenerational effects, DNA repair genes

## Abstract

The study of the genetic basis of the manifestation of radiation-induced effects and their transgenerational inheritance makes it possible to identify the mechanisms of adaptation and possible effective strategies for the survival of organisms in response to chronic radioactive stress. One persistent hypothesis is that the activation of certain genes involved in cellular defense is a specific response of the cell to irradiation. There is also data indicating the important role of transposable elements in the formation of radiosensitivity/radioresistance of biological systems. In this work, we studied the interaction of the systems of *hobo* transposon activity and DNA repair in the cell under conditions of chronic low-dose irradiation and its participation in the inheritance of radiation-induced transgenerational instability in *Drosophila*. Our results showed a significant increase of sterility and locus-specific mutability, a decrease of survival, fertility and genome stability (an increase the frequency of dominant lethal mutations and DNA damage) in non-irradiated F_1_/F_2_ offspring of irradiated parents with dysfunction of the *mus304* gene which is responsible for excision and post-replicative recombination repair and repair of double-stranded DNA breaks. The combined action of dysfunction of the *mus309* gene and transpositional activity of *hobo* elements also led to the transgenerational effects of irradiation but only in the F_1_ offspring. Dysfunction of the genes of other DNA repair systems (*mus101* and *mus210*) showed no visible effects inherited from irradiated parents subjected to *hobo* transpositions. The *mei-41* gene showed specificity in this type of interaction, which consists in its higher efficiency in sensing events induced by transpositional activity rather than irradiation.

## Introduction

Ionizing radiation induces biological and genetic effects that have been the subject of detailed study for many years. One of the most important cellular systems responding to irradiation is the DNA repair system which is actively involved in the elimination of DNA damages even before they turn into mutational events (Bregliano et al., 1995). When the cell is irradiated, an increased activity of transposable elements (TEs) associated with stress resistance genes, in particular, DNA repair genes, is observed. TEs are a movable part of the genome and DNA fragments that can move around the genome and destabilize it (Finnegan, 1989; Kaufman and Rio, 1992; Jurka et al., 2007; McCullers and Steiniger, 2017). To date, a connection has been established between *P* elements and repair processes which is described details in Banga et al. (1991) and Yushkova and Zainullin (2016). The *P* transposon acting as a “model” intracellular factor is capable of destabilizing the genome only in a certain genetic P-M system of hybrid dysgenesis (HD) (Kidwell et al., 1977). HD is a syndrome that manifests in offspring in the form of an increased level of gene mutations, chromosomal aberrations, chromosome non-disjunction during meiosis, and sterility. HD occurs with certain crossing combinations. As a result of such crosses, inactive transposons (*P* and *hobo*) of males of strong P and H strains become active after crossing with females of M and E strains which lack the repressor protein of transposition (Kidwell et al., 1977; Bazin and Higuet, 1996). Entering the cytoplasm lacking the repressor protein of transposition, *P* and *hobo* elements encode the enzyme (transposase) of their own transposition which lead to DNA damage and genetic breakdowns (Bazin et al., 1999). As a result, dysgenic events occur in the offspring in early ontogeny, causing atrophy of the reproductive organs, a decrease of fertility and survival at the later stages of their development (Niki and Chigusa, 1986). The presence of certain properties inherited through the maternal line is a key characteristic of the “cytotype” and determines the level of functional activity of transposons responsible for the manifestation of HD (Engels, 1981). A feature of *P* and *hobo* elements is their wide distribution and a high level of diversity in various organisms, including humans (Menzel et al., 2014). Unlike other TEs (retrotransposons that move by the “copy-paste” mechanism and do not break the integrity of the DNA structure), transposons are able to move through the genome using the “cut-paste” mechanism and lead to the formation of double-stranded DNA breaks (DSBs) (Sobels and Eeken, 1981; Finnegan, 1989; Kaufman and Rio, 1992).

Recently, there has been a growing interest in studies of the behavior of *hobo* elements which not only can be massively activated under conditions of classical dysgenic crossing [H-males (with *hobo* transposons) × E-females (without *hobo*)] (Blackman et al., 1987) but they can also be active in non-genetic systems, i.e., under conditions of interlinear (between H-males and H-females) and reciprocal (between H-females and E-males) crosses (Bazin and Higuet, 1996; O’Hare et al., 1998; Zakharenko et al., 2006; Yushkova, 2019). The *hobo* transposon of *D. melanogaster* belongs to the *Ac* elements of the *hAT* superfamily (Loreto et al., 2018). It is active in the germ line and somatic tissue cells (Calvi and Gelbart, 1994; O’Hare et al., 1998; Zakharenko et al., 2006) and also is sensitive to radiation exposure (Ivashenko and Grishaeva, 2002; Yushkova and Zainullin, 2014). The full-length *hobo* element (∼ 3 kb) has an open reading frame (ORF) encoding transposase and short terminal inverted repeats (TIRs) 12 bp in size, forms duplications at the insertion site of 8 bp (Loreto et al., 2018). Many studies have shown that *Drosophila* genomes are loaded the defect or truncated *hobo* copies (up to 1.5 kb) which have a high similarity to a transcriptionally active canonical *hobo* transposons (Torres et al., 2006; Ortiz and Loreto, 2008; Deprá et al., 2009). Such copies move with high frequency and may play a key role in the formation of dysgenic status in animals (Bazin and Higuet, 1996). Canonical *hobo* elements have ORF or only part of it (due to internal deletions of *hobo* copies) and TIRs (Loreto et al., 2018). Transposons of the *hAT* superfamily have some structural and functional homology with full-length human transposons (*Charlie 1-8*, *Cheshire*, *Zaphod*, and *MER69*) which account for 1.55% (195 Mbp) of the total genome (Lander et al., 2001). In general, transposons are not active in the human genome because they are not autonomous (McCullers and Steiniger, 2017). However, there is information confirming the presence of active *THAP9* transposons in the human genome capable to transpositions, like the *P* elements of *D. melanogaster* (Majumdar et al., 2013; Majumdar and Rio, 2015).

This article is a continuation of the study of the mechanisms of interaction between the systems of transposition of TEs as components of epigenetic regulation and DNA repair in the cell under conditions of chronic radiation exposure (Yushkova and Zainullin, 2016). We have established that there is a specificity of turning-on of certain repair processes and their genes as a result of a particular effect (transposition of *P* elements or irradiation). The “universality” of some repair genes functioning upon the simultaneous action of external (irradiation) and intracellular (*P* elements) factors has also been discovered. The question remains to what extent this cell response depends on the “transposon-specificity” in this type of interaction. One of the possible approaches to solving this problem is to study the involvement of DNA repair genes in the processes of DNA damage repair induced by the transpositions of *hobo* elements under conditions of chronic irradiation.

The ability of transposons to cause such serious damage as (DSBs) attracts a significant interest in the problem of radiation-induced activation of transposition systems under conditions of impaired DNA repair. Given the fact that dysfunction of certain repair genes can lead to the inheritance of genetic effects by the offspring of irradiated parents (Yushkova, 2020), it would be interesting to consider how the interaction of the repair genes and transpositional activity of TEs affect the transgenerational transmission of radiation-induced genome instability. Moreover, the aforementioned studies on the transgenerational effects of irradiation have been mainly carried out on animals using high doses of irradiation (Wiley et al., 1997; Barber et al., 2002; Sarapultseva and Malina, 2009; Sarapultseva and Bychkovskaya, 2010; Sarapultseva and Gorski, 2013; Gomes et al., 2015; Sarapultseva and Dubrova, 2016; Lecomte-Pradines et al., 2017; Fuller et al., 2019; Hancock et al., 2019). Therefore, from this point of view, it is also important to consider whether parental radiation exposure affect cytogenetic and epigenetic traits inherited by the offspring of irradiated parents.

## Materials and Methods

### Fly Strains

The strains were obtained from the Bloomington Drosophila Stock Center and characterized using molecular (PCR analysis) and genetic (instability of the *mini-white* locus) methods, which made it possible to determine the content of full-length *hobo* copies in their genomes (Galindo et al., 1995; Bazin and Higuet, 1996; Zakharenko et al., 2006; Yushkova and Zainullin, 2014).

#### Strains With Functional *hobo* Transposons

*OR*^*S*^ (genotype: *C^1^DX, y^1^f^1^;OR*) is a subline of the H-cytotype with attached X-chromosomes. It is derived from the wild-type *Oregon-R* (*OR*) strain containing full functional copies of the *hobo* element in the genotype (Galindo et al., 1995). It was created by crossing three females of the *C^1^DX, y^1^f^1^/Y* strain with three *OR* males followed by backcrossing their offspring (females) with *OR* males, for 8 generations (Margulies, 1990). It is capable of high induction of sterility and is subject to instability of the *mini-white* locus at a frequency of ∼9%. PCR analysis revealed the presence of full-length *hobo* elements in its genotype. We used this strain as an inducer strain of the H-cytotype.

#### Strains Without *hobo* Transposons

*HiR*^*S*^ (genotype: *C^1^DX*, *y^1^f^1^*; *HiR*) is a subline of the E-cytotype that does not have *hobo* transposons. It was obtained according to the same crossing scheme as the *OR*^*S*^ subline (from the wild-type *Hikone-R* strain which does not contain *hobo* elements (Galindo et al., 1995). We used it in crosses as a reactive (sensitive to *hobo* transposition) E-cytotype strain that is not subject to mutability in the *mini-white* locus.

*mei-41* (genotype: *mei-41^*RT*1^ f^1^/FM7a*) is a strain of the E-cytotype with a mutation in the *mei-41* gene involved in the DNA damage sensing, post-replicative DNA repair, meiotic recombination and the G2/M checkpoint (Hari et al., 1995; Laurencon et al., 2003). The *mei-41* gene is also involved in maintaining chromosome stability upon transposon activity (Koromyslov et al., 2003). We used this strain as a conditional positive control. It is not subject to mutability in the *mini-white* locus.

*mus210^*G*1^* (genotype: *mus210^*G*1^/CyO*) are strain of the E-cytotype with a mutation in the *mus210* gene, which participates in excision repair (Sekelsky et al., 1998). The presence of mutations in genes responsible for the excision repair in the genome does not impair the stability of chromosomes experiencing transposon activity (Chmuzh et al., 2007). We used this strain as a conditional negative control. When it was crossed with the *haw* strain, no mutability in the *mini-white* locus was found.

*mus101^*D*1^* (genotype: *w^1^mus101^*D*1^*) is a strain of the E-cytotype with a mutation in the *mus101* gene involved in the control of the cell cycle and non-recombination post-replicative DNA repair (de Buendßa et al., 2003; Choi et al., 2017). When it was crossed with the *haw* strain, no mutability in the *mini-white* locus was found.

*mus304^*D*1^* (genotype: *st^1^mus304^*D*1^/TM3,Sb^1^Ser^1^*) is a strain of the E-cytotype with a mutation in the *mus304* gene involved in the cell cycle control, in post-replicative DNA repair and recombination, DSB repair and excision repair of pyrimidine dimers (Harris and Boyd, 1993; Brodsky et al., 2000). It is not subject to mutability in the *mini-white* locus.

*mus309^*D*3^* (genotype: *mus309^*D*3,ry^/CyO*) is an E-strain with a mutation in the *mus309* gene involved in the DSB repair system (Portin, 2010). When it was crossed with the *haw* strain, no mutability in the *mini-white* locus was found.

#### Test Strains

*haw* (genotype: *ywH^*w+,haw*1^*) is an E-strain carrying two genetically engineered marker elements *hobo*(*w*^+^) on the X chromosome with an inserted *mini-white* reporter gene which is responsible for the orange color of the eyes. A change in the eye color in males (less brightly colored eyes) indicates the excision of one of the *hobo*(*w*^+^) elements (Calvi and Gelbart, 1994). This strain was kindly provided by B. R. Calvi (University of Philadelphia, Philadelphia, PA, United States). We used this strain as a way to quantify the activity of transposase of *hobo* elements.

*ywf/mei-41* (genotype: *mei-41/y&C^1^DX;y^1^w^1^f^1^/y*) has attached X chromosomes and traits *y* (*yellow –* yellow body), *f* (*forked –* underdeveloped bristle), and *w* (*white –* white eyes). We used this strain in individual crosses to determine mutability in the *mini-white* locus.

### Experimental Design

The analysis of the transpositional activity of *hobo* elements under conditions of impaired protective intracellular processes was carried out by inducing crosses. To obtain F_1_ offspring, mass crosses of males of the *OR*^*S*^ strain carrying full-length copies of *hobo* elements with females of the E-strains *HiR*^*S*^, *mei-41*, and *mus*-groups were carried out. To obtain F_2_ offspring, F_1_ specimens were individually crossed with the studied E-strains. The offspring from these crosses were conventionally designated as variants *HiR*[*hobo*+], *mus*[*hobo*+], and *mei-41*[*hobo*+]. As a control, we used specimens obtained by crossing *HiR*^*S*^ males without *hobo* sequences with females of *HiR*^*S*^, *mus101^*D*1^*, *mus210^*G*1^*, *mus304^*D*1^*, *mus309^*D*3^*, and *mei-41* strains. The control variants received the following designations: *HiR*[*hobo-*], *mus*[*hobo-*] and *mei-41*[*hobo-*]. A total of 24 crossing variants were analyzed, including irradiated variants.

### Irradiation Conditions

Chronic irradiation of parents was performed from a ^226^Ra source (56 mGy/h) at a dose rate of 0.42 mGy/h. The cumulative dose was 120.9 mGy (from the egg stage to the imago, 12 days). Thermoluminescent dosimeters “DTU-1” with detectors “DTG-4” (“Dosa,” Russia) were used to measure the irradiation doses. Background specimens underwent the same procedure without irradiation. The parental generation of all variants was maintained under strictly controlled laboratory conditions with a photoperiod of 12 h light/12 h darkness at 25 ± 0.1°C and 60% humidity on a standard sugar-yeast medium (Ashburner, 1989).

### Analysis of Dominant Lethal Mutations

The analysis of dominant lethal mutations (DLMs) was carried out according to the generally accepted method (Watti and Tikhomirova, 1976; Aslanian et al., 1994). The F_1_/F_2_ offspring were crossed with virgin females of the corresponding strain. The final progeny at the egg stage was evaluated for the presence of colored (brown) embryos. These changes called DLMs are caused by genetic abnormalities, which are large chromosomal rearrangements (Watti and Tikhomirova, 1976). To increase the visibility of the laid eggs, we used a sugar-yeast nutrient medium supplemented with activated carbon (Richardson and Kojima, 1965). The DLM frequency was calculated as a ratio of the number of later lethals to the total number of laid embryos. Each variant of the experiment was conducted in three replicates.

### Survival Analysis

From each variant, we selected F_1_/F_2_ males (100 specimens) within 24 h after hatching of the imago. The studied variants were maintained under constant conditions on a depleted medium (sugar-agar) at 25 ± 0.1°C and 60% humidity with a 12 h light/12 h darkness cycle (Ashburner, 1989). Specimens were transferred to a fresh medium twice a week. The survival rate of specimens was assessed daily. Each variant of the experiment was conducted in three replicates.

### Fertility Analysis

From each F_1_/F_2_ variant, we selected 50 control and experimental males of the same age and crossed them with virgin females of the corresponding genotypes at a 1:3 ratio. For 10 days, the number of embryos laid by females per day was counted. Tubes with counted eggs were stored at 25 ± 0.1°C and 60% humidity with a 12 h light/12 h darkness photoperiod until the pupation stage, after which the pupae were counted. For a more accurate visual registration of the laid eggs, a nutrient sugar-yeast medium supplemented with activated carbon was used (Richardson and Kojima, 1965). Fertility was calculated as the average number of pupae per average number of embryos per female. Each variant of the experiment was conducted in three replicates.

### Sterility Analysis

From each F_1_/F_2_ variant, males of the same age (100 specimens) were selected. They were crossed with wild-type E-females (♂*HiR*[*hobo-*] or ♂*HiR*[*hobo*+] × 3♀♀*HiR*^*S*^) and mutant genotype females (♂*mus*(*mei-41*)[*hobo-*] or *mus*(*mei-41*)[*hobo*+] × 3♀♀*mus*(*mei-41*)). Sterility (GD) was assessed using the method of gonadal atrophy. The presence [unilateral (GD1) and bilateral (GD0)] of hybrid females of each F_1_/F_2_ variant was determined by dissecting the abdomen. After emergence from puparia, females were maintained on a nutrient medium for 3 days until the full maturation of the gonads. The GD sterility was calculated using the formula: GD (%) = GD0 (%) + 1/2 GD1 (%) (Marin et al., 2000). Each variant of the experiment was conducted in three replicates.

### Analysis of Mutability of the *mini-white* Locus

The parameter “mutability of the *mini-white* locus” makes it possible to assess the activity of transposase, an enzyme responsible for the transpositions of *hobo* transposon, in somatic cells (Bazin and Higuet, 1996). For this, a series of successive crosses of experimental F_1_/F_2_ males with *haw* and *ywf/mei-41* test strains were carried out. The offspring were screened for specific specimens with a *yellow* (yellow body), *forked* (underdeveloped bristle) phenotype and mosaic eye pigmentation that occurs during somatic transposition or excision of *hobo*(*w*+) during development. This test allows the assessment of excision (Me) and transposition (Mt) of two *hobo*(*w*+) reporter *hobo* elements and tracking the activity of *hobo* elements in the tested males through the transposition of *hobo*(*w*+) from the X chromosome to autosomes (Calvi et al., 1991; Bazin and Higuet, 1996). The appearance in the offspring of males with lightened eyes (less-colored-eyes) indicates *hobo*(*w*+) excision. No excision was found in our study. While the appearance of females with pigmented eyes indicates an autosomal transposition of *hobo*(*w*+) (Mt). The level of transpositions (%Mt) was assessed by the number of hybrid males that had at least one transposition event in their offspring. Each variant of the experiment was conducted in three replicates.

### DNA Damage Analysis

DNA damage in neuroblasts and midgut cells of F_1_/F_2_ third instar larvae was assessed using the Comet assay (Yushkova and Zainullin, 2016). The cell suspension was obtained by treating somatic tissues to a collagenase solution (type IV, 0.5 mg/ml PBS) at 37°C. The obtained cells of the nerve ganglia and midgut (10 μl) were mixed with 0.5% low melting point agarose (100 μl) and applied on glass slides coated with a layer of 1% normal melting point agarose. The slides were kept in a cold environment (4°C) for 20 min and subjected to further lysis (2.5 Ì NaCl, 10 ìÌ Na_2_EDTA, 20 ìÌ Tris–HCl, 1% Triton X-100, pH 10.0) at 4°C. Electrophoresis was performed (at 30 mA, 0.7 V/cm, 4°C, 20 min) in a cooled (4°C) neutral buffer (0.9 M Trizma-Base, 0.9 M Boric acid, 20 mM Na_2_EDTA, pH 8.2). The neutral version of the method assesses predominantly DSBs (Olive and Banath, 2006). The preparations were fixed in 70% ethanol for 15 min, dried and stained with the SYBR Green I solution (0.2 μL/ml in TE buffer, pH 7.5) (DNA synthesis, Russia). The images of “comets” were analyzed using an Infiniti XS-148 FS fluorescence microscope and processed using the CometScore^TM^ software (version 1.5, TriTek Corp., United States)^1^. The DSBs were assessed by the Olive tail moment (OTM). OTM is the product of the distance from the center of the head to the density center of the comet’s tail multiplied by tDNK[%] (concentration (%) of DNA in the tail of the “comet”) (Olive, 1999). For each experimental point (10 specimens), 3 slides were taken. On each slide, 50 cells were counted. Given into account three replicates of each variant of the experiment, 450 cells were analyzed per variant, per generation.

We used the following reagents: sodium chloride (NaCl), agarose, triton-X, boric acid, tris hydrochloride (Tris–HCl) (PanReac, Spain), collagenase type IV, phosphate buffered saline (PBS), tris(hydroxymethyl)aminomethane (Trizma Base) (Sigma, CIIIA), disodium EDTA (Na2EDTA) (AppliChem, Germany).

### PCR Analysis

PCR analysis was performed as described in Yushkova and Zainullin (2014). DNA was isolated from the imago using the Diatom^TM^ DNA Prep 200 kit (IzoGen, Russia). The primers for the open reading frame of the full-length *hobo* element were 5′-GCGCCATACATAATGATTG-3′ and 5′-CTATTGCGAGTTGTTTAG-3′ (Zakharenko et al., 2006). The DNA concentration was measured using a Qubit^®^ fluorometer (Invitrogen, Waltham, MA, United States). Amplification of the isolated DNA was carried out with the addition of GenPak^®^ PCR MasterMix Core reagents using an ESCO Swift Mini Pro thermal cycler (ESCO, Singapore). PCR reaction parameters: initial denaturation at 94°C for 3 min; 35 cycles of denaturation at 94°C for 30 s, annealing at 54°C for 30 s, synthesis at 72°C for 100 s; final elongation at 72°C for 5 min. As a control, deionized water was used. PCR products were stained with the SYBR Green I dye (DNA synthesis, Russia) in 1.3% agarose gel and separated by horizontal electrophoresis. Electrophoretograms were visualized using an ECX-15.M transilluminator (Vilber Lourmat, France) and a Gel Imager video system (DNA-Technology, Russia).

### Statistical Analysis

Three independent experiments were carried out, the results of which were summarized and presented as a mean value and the corresponding standard error of the mean (SEM). Statistical analysis of differences between the mean values of the parameters (the frequency of DNA damage and DLMs, the level of sterility and Mt) of the experimental variants were determined by the Student’s *t*-test. The obtained data were processed using the Statistica program (version 7.0.61.0, StatSoft, Inc., United States). The significance of differences of the results of the fertility analysis was assessed by the Chi-square test in the R program. To compare the statistical differences in survival functions and median lifespan, as well as maximum lifespan between the control and experimental groups, the Kolmogorov-Smirnov, log-rank and Wang-Allison tests were used, respectively (Mantel, 1966; Breslow, 1970; Fleming et al., 1980; Han et al., 2016). Data were processed using the Online Application for Survival Analysis 2 (OASIS 2)^2^.

## Results

### Activity of *hobo* Transposons

GD-sterility is one of the important traits of the manifestation of the H-E system of the HD which is based on the high transposition activity of *hobo* elements (Bazin and Higuet, 1996). The results obtained showed that increased GD-sterility is characteristic for all offspring obtained by crosses with *OR*^*S*^ males (Table 1). On the contrary, we did not observe offspring with gonadal atrophy when crossed with male *HiR*^*S*^. It follows from this that the observed sterility, albeit indirectly, testifies to the transpositional activity of *hobo* elements. The strains *HiR*^*S*^, *mei-41*, and *mus304^*D*1^* were the most sensitive to *hobo* transpositions. The offspring with dysfunction of the *mei-41* and *mus304* genes exhibited high sterility (*p* < 0.05) independent of parental exposure. For this parameter, transgenerational effects of irradiation (*p* < 0.05) were observed in F_1_/F_2_ offspring with normal functioning of repair genes (*HiR*^*S*^).

**TABLE 1:** Effect of a chronic irradiation in low doses and induction of the *hobo* elements on the sterility of *D. melanogaster* females F_1_/F_2_ with dysfunction of the repair genes.

Genotype	Variant	F_1_			F_2_		
		GD,% (N)	GD1,% (n)	GD0,% (n)	GD,% (N)	GD1,% (n)	GD0,% (n)
*HiR*^*S*^	*hobo*+	44.3 ± 3.4	0.33 ± 0.1	44.1 ± 2.6	32.1 ± 2.6*	3.65 ± 1.1	30.3 ± 2.5*
		(607)	(2)	(268)	(712)	(26)	(216)
*HiR*^*S*^	[*hobo+*] + γ	68.7 ± 4.1*^*a*^*	0.27 ± 0.1	68.6 ± 3.9*^*a*^*	44.7 ± 2.8*^*a*^*	3.47 ± 1.1	43.0 ± 2.8*^*a*^*
		(737)	(2)	(506)	(634)	(22)	(274)
*mei-41*	*hobo*+	50.1 ± 3.8	0	50.1 ± 3.8	30.1 ± 3.1*	9.28 ± 2.0	25.5 ± 3.0*
		(698)	(0)	(350)	(517)	(47)	(132)
*mei-41*	[*hobo*+] + γ	54.6 ± 4.3^#^	0.84 ± 0.1*	54.2 ± 3.7^#^	29.7 ± 2.8*	7.46 ± 1.2*	26.0 ± 2.5^#^
		(714)	(6)	(387)	(496)	(37)	(129)
*mus101*	*hobo*+	19.6 ± 2.4	8.52 ± 1.9*^*c*^*	15.3 ± 2.1*^*c*^*	20.3 ± 2.1	7.16 ± 1.7	16.7 ± 2.0^#^
		(516)	(44)	(79)	(503)	(36)	(84)
*mus101*	[*hobo*+] + γ	28.1 ± 2.2*^*b*^*	3.67 ± 1.1*^*c*^*	26.3 ± 2.3*^*e*^*	23.6 ± 2.4*^*d*^*	9.49 ± 1.7	18.9 ± 2.1
		(544)	(20)	(143)	(516)	(49)	(98)
*mus210*	*hobo*+	12.8 ± 2.0*^*c*^*	6.21 ± 1.8*^*c*^*	9.66 ± 1.9*^*c*^*	14.5 ± 3.2*^*c*^*	10.0 ± 2.6	9.50 ± 2.9*^*c*^*
		(611)	(38)	(59)	(549)	(55)	(52)
*mus210*	[*hobo*+] + γ	15.6 ± 2.1*^*c*^*	5.74 ± 1.7*^*c*^*	12.7 ± 2.0*^*c*^*	10.8 ± 2.1*^*c*^*	7.10 ± 1.1	7.26 ± 1.3*^*c*^*
		(575)	(33)	(73)	(606)	(43)	(44)
*mus304*	*hobo*+	55.8 ± 4.6*^*f*^*	4.40 ± 1.5*^*c*^*	53.6 ± 4.2	54.9 ± 3.1*^*c*^*	8.86 ± 1.8	50.5 ± 2.8*^*c*^*
		(704)	(31)	(378)	(711)	(63)	(359)
*mus304*	[*hobo*+] + γ	51.8 ± 5.2	6.40 ± 1.9*^*c*^*	48.6 ± 4.6 *^*f*^*	42.8 ± 2.8*^*f*^*	7.19 ± 1.6	39.2 ± 2.4*^*g*^*
		(593)	(38)	(288)	(681)	(49)	(267)
*mus309*	*hobo*+	17.6 ± 2.3*^*c*^*	4.34 ± 1.7*^*c*^*	15.4 ± 2.5*^*c*^*	17.1 ± 1.2*^*c*^*	8.00 ± 2.1	13.1 ± 2.6*^*c*^*
		(507)	(22)	(78)	(487)	(39)	(64)
*mus309*	[*hobo*+] + γ	24.2 ± 2.9*^*c*^*	6.36 ± 1.8*^*c*^*	21.0 ± 2.7*^*c*^*	19.7 ± 2.0*^*c*^*	7.72 ± 1.8	15.8 ± 2.2*^*c*^*
		(519)	(33)	(109)	(544)	(42)	(86)

The differences are statistically significant at *p < 0.05 between F_1_ and F_2_; ^#^*p* < 0.05 with normal repair genes (*HiR*^*S*^); *^*a*^p* < 0.05 with own control (without (*hobo-*) and with (*hobo*+) transposons induction) and between F_1_ and F_2_; *^*b*^p* < 0.05 with own control, *HiR*^*S*^ and positive control (*mei-41*); ^*c*^*p* < 0.05 with *HiR*^*S*^ and positive control (*mei-41*); *^*d*^p* < 0.05 with *HiR*^*S*^ and negative control (*mus210*), between F_1_ and F_2_; *^*e*^p* < 0.05 with *HiR*^*S*^, positive control (*mei-41*) and negative control (*mus210*); *^*f*^p* < 0.05 with negative control (*mus210*); *^*g*^p* < 0.05 with positive control (*mei-41*) and negative control (*mus210*); errors indicate the standard error of the mean (±SEM) for *n* = 3 independent experiments. GD, GD1 and GD0 - explanations in the section “Materials and methods”; N – total number females; n - number females with gonadal atrophy.

**FIGURE 1:**
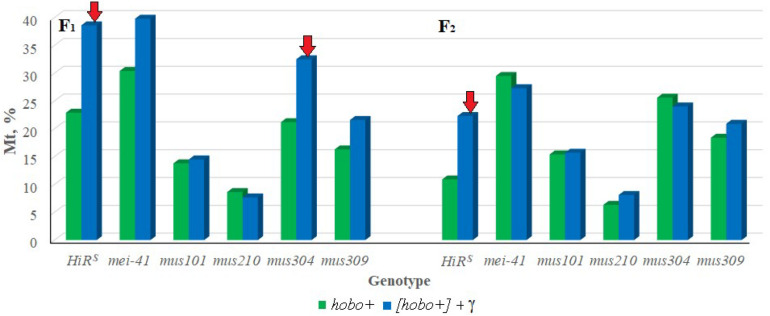
Effect of a chronic irradiation in low doses and induction of the *hobo* elements on the autosomal transposition *hobo*(*w*^+^) of *D. melanogaster* females F_1_/F_2_ with normal repair genes (*HiR*^*S*^) and dysfunction of the repair genes (*mei-41*, *mus101*, *mus210*, *mus304* and *mus309*). Red arrow – statistically significant (*p* < 0.05–0.01) transgenerational effects of irradiation in individuals with dysfunction of the repair genes in the presence transposition of *hobo* elements.

To confirm the *hobo* activity, we also used a specific test for this based on the detection of autosomal excisional and transpositional activity of the constructed marker *hobo*(*w*+) elements in the offspring from crosses with males of the studied genotypes with *haw* females (Calvi et al., 1991; Calvi and Gelbart, 1994). Their activity depends on the transposase encoded by full-length *hobo* elements presumably present in the paternal genotypes. In the presence of transposase, *hobo*(*w*+) elements manifest their activity in the form of excisions and transpositions. In our study, excisions of *hobo*(*w*+) elements were not revealed, but were found their transpositions (Mt). The F_1_/F_2_ offspring selected as a result of crossing with *OR*^*S*^ are characterized by high activity of *hobo* elements (*p* < 0.05–0.01). The levels of their GD sterility and Mt varied within 12.8–55.8 and 6.3–30.4% (in the offspring of non-irradiated parents) and 10.8–68.9 and 8.1–39.8% (in the offspring of irradiated parents), respectively. This proves the presence of statistically significant transposition activity of *hobo* elements in the studied genotypes. Transgenerational inheritance of different levels *hobo* activity (from low to high) is retained in all offspring of all genotypes independent of parental exposure. Statistically significant (*p* < 0.01) transgenerational effects of irradiation were found in F_1_/F_2_ offspring with normal functioning of repair genes (*HiR*^*S*^) and F_1_/F_2_ offspring with dysfunction of the *mus304* gene undergoing *hobo* transposition (Figure 1).

All experimental variants were tested for the content of full-length *hobo* copies in their genotypes. PCR analysis showed the presence of full-length *hobo* transposons only in the offspring with *hobo* activity.

### DLM

**FIGURE 2:**
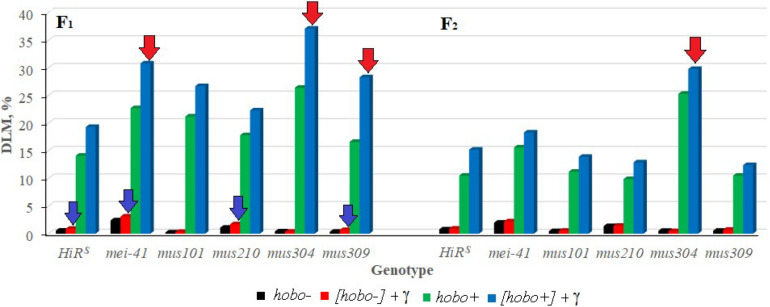
Effect of a chronic irradiation in low doses and induction of the *hobo* elements on the DLM frequency of *D. melanogaster* individuals F_1_/F_2_ with normal repair genes (*HiR*^*S*^) and dysfunction of the repair genes (*mei-41*, *mus101*, *mus210*, *mus304*, and *mus309*). Blue arrow – statistically significant (*p* < 0.05–0.01) transgenerational effects of irradiation in individuals with dysfunction of the repair genes and without induction of *hobo* elements; red arrow – statistically significant (*p* < 0.05–0.01) transgenerational effects of irradiation in individuals with dysfunction of the repair genes in the presence transposition of *hobo* elements.

It was shown that disturbances in post-replicative (gene *mei-41*), excisional (gene *mus210*) repair, and DNA DSB repair (gene *mus309*) significantly affect (*p* < 0.05–0.01) the transmission of DLM frequency to non-dysgenic F_1_ offspring after irradiation of their parents (Figure 2). In the presence of *hobo* elements, the frequency of radiation-induced DLM increases (*p* < 0.01) in offspring with dysfunction of the *mei-41*, *mus304*, and *mus309* genes. The inheritance of an increased level of DLM by subsequent generations depends on the dysgenic status of animals and the functional state of the *mus304* gene in their genome. Dysfunction of the *mus304* gene leads to significant (*p* < 0.01) transgenerational effects of parental irradiation in F_2_ offspring undergoing transposition of *hobo* elements.

### DNA Damage

Our results indicate a significant increase in the frequency of DSBs in somatic cells of substantially all dysgenic F_1_ offspring (except for specimens with dysfunction of the *mus101* and *mus210* genes) (Table 2). Transgenerational transmission of DSBs formed as a result of *hobo* activity was observed in the F_1_/F_2_ offspring of the *HiR*^*S*^, *mei-41*, and *mus304^*D*1^* genotypes (*p* < 0.01). Parental radiation exposure enhances the *hobo* activity, leading to the formation of DNA damage in the cells of the F_1_ offspring of the *HiR*^*S*^ strain and strains with disfunction of the *mei-41* (only in midgut cells), *mus304*, and *mus309* (in all tissues) genes (*p* < 0.05–0.01). The transgenerational effect of irradiation is retained in all studied somatic cells in the F_2_ offspring with disfunction of the *mus304* (*p* < 0.01), *mei-41* (only in brain cells, *p* < 0.05) and *mus309* (only in midgut cells, *p* < 0.05) genes. Disgenic offspring with disfunction of the *mus304* gene after parental radiation exposure (as well as the positive control – *mei-41*) had higher OTM values.

**TABLE 2:** Radiation- and *hobo*-induced DSB DNA (OTM, arb. units) in midgut and brain cells of *D. melanogaster* larvae.

Genotype	Variant	F_1_				F_2_			
		Brain (OTM)	Brain (dOTM)	Midgut (OTM)	Midgut (dOTM)	Brain (OTM)	Brain (dOTM)	Midgut (OTM)	Midgut (dOTM)
*HiR*^*S*^	*hobo*-	0.56 ± 0.09		0.54 ± 0.08		0.59 ± 0.09		0.41 ± 0.06	
*HiR*^*S*^	[*hobo-*] + γ	0.96 ± 0.19*	71.4	0.79 ± 0.07	46.3	0.73 ± 0.11	23.7	0.88 ± 0.12*	114
*HiR*^*S*^	*hobo*+	1.28 ± 0.13		1.02 ± 0.11		0.94 ± 0.11		1.14 ± 0.21	
*HiR*^*S*^	[*hobo*+] + γ	1.89 ± 0.24**	47.6	1.61 ± 0.17**	57.8	1.33 ± 0.21*	41.5	1.28 ± 0.18	12.8
*mei-41*	*hobo-*	0.71 ± 0.12		0.63 ± 0.11		0.76 ± 0.11		0.59 ± 0.09	
*mei-41*	[*hobo-*] + γ	1.14 ± 0.15*	60.6	0.98 ± 0.08	55.5	0.81 ± 0.11	6.6	0.87 ± 0.0847.4	
*mei-41*	*hobo*+	2.13 ± 0.23^*aa*^		1.96 ± 0.20^*aa*^		1.99 ± 0.18^*aa*^		1.89 ± 0.13^*aa*^	
*mei-41*	[*hobo*+] + γ	2.36 ± 0.25^*aa*^	10.8	2.68 ± 0.24^**^,^*aa*^	36.7	2.29 ± 0.16^*^,^*aa*^	15.1	2.03 ± 0.19^*aa*^	7.4
*mus101*	*hobo-*	0.37 ± 0.04^*bc*^		0.51 ± 0.06		0.48 ± 0.05		0.93 ± 0.12^*a*,*b*^	
*mus101*	[*hobo-*] + γ	0.44 ± 0.05^*a*,*bb*^	18.9	0.67±0.07^*c*^	31.4	0.81 ± 0.09*	68.7	0.86 ± 0.11	–7.5
*mus101*	*hobo*+	0.68 ± 0.08^*aa*,*bb*^		0.77 ± 0.09		0.64 ± 0.09^*bb*^		0.55 ± 0.07^*aa*,*bb*^	
*mus101*	[*hobo*+] + γ	0.86 ± 0.09^*aa*,*bb*^	26.5	0.92 ± 0.09^*aa*,*bb*^	19.5	0.78 ± 0.06^*aa*,*bb*^	21.8	0.80 ± 0.08^*a*,*bb*^	45.4
*mus210*	*hobo-*	0.86 ± 0.10		0.74 ± 0.07		0.69 ± 0.05		0.83±0.09^*a*^	
*mus210*	[*hobo-*] + γ	1.18 ± 0.14*	37.2	1.03 ± 0.13	39.2	0.85 ± 0.09	23.2	0.94 ± 0.12	13.2
*mus210*	*hobo*+	0.94±0.11^*a*^		0.82 ± 0.11		1.02±0.13^*c*^		0.79±0.08^*a*^	
*mus210*	[*hobo*+] + γ	0.91 ± 0.13^*aa*^	–3.2	0.74 ± 0.08^*aa*^	–9.7	0.77 ± 0.06^*aa*^	–24.5	0.56 ± 0.06^*aa*^	–29.1
*mus304*	*hobo-*	0.46 ± 0.04		0.69 ± 0.08		0.69 ± 0.07		0.81±0.07^*a*^	
*mus304*	[*hobo-*] + γ	0.57 ± 0.05^*a*,*b*,*cc*^	23.9	0.53 ± 0.04,^*b*,*c*^	–23.2	0.88 ± 0.12	27.5	1.27 ± 0.16^*^,^*a*,*b*,*c*^	56.7
*mus304*	*hobo*+	0.97 ± 0.15^*a*,*c*^		1.16 ± 0.17^*bb*^		1.12 ± 0.14^*bb*^		1.44 ± 0.51^*a*,*b*,*cc*^	
*mus304*	[*hobo*+] + γ	1.92 ± 0.26*^*^,^*bb*,*cc*^	97.9	2.14 ± 0.39*^*^,^*aa*,*b*,*cc*^	84.5	1.81 ± 0.22*^*^,^*a*,*b*,*cc*^	61.6	2.01 ± 0.24*^*^,^*aa*,*cc*^	39.6
*mus309*	*hobo-*	0.51 ± 0.06		0.48 ± 0.09		0.61 ± 0.09		0.81±0.08^*a*^	
*mus309*	[*hobo-*] + γ	0.68 ± 0.07^*b*,*cc*^	33.3	0.74 ± 0.09	54.1	0.94 ± 0.11*	54.1	0.81 ± 0.130	
*mus309*	*hobo*+	0.93 ± 0.14^*a*,*b*^		0.82 ± 0.12^*bb*^		0.87 ± 0.13		0.63 ± 0.17^*aa*^	
*mus309*	[*hobo*+] + γ	1.24 ± 0.21^*^,^*a*,*bb*^	33.3	1.59 ± 0.14^**bb,*cc*^	93.9	1.01±0.15^*a*^	16.1	0.99 ± 0.19*	57.1

The differences are statistically significant at *p < 0.05, **p < 0.01 with own control, without (*hobo-*) and with (*hobo*+) transposons induction; *^*a*^ p* < 0.05, *^*aa*^ p* < 0.01 with genotype without mutations (*HiR*^*S*^); *^*b*^p* < 0.05, *^*bb*^p* < 0.01 with positive control (*mei-41*); ^*c*^*p* < 0.05, *^*cc*^p* < 0.01 with negative control (*mus210*); dOTM – the differences in average OTM values; errors indicate the standard error of the mean (±SEM) for *n* = 3 independent experiments.

### Fertility

**FIGURE 3:**
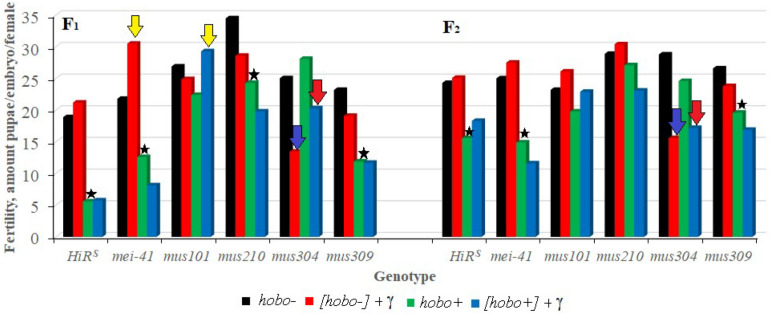
Effect of a chronic irradiation in low doses and induction of the *hobo* elements on the fertility of *D. melanogaster* females F_1_/F_2_ with normal repair genes (*HiR*^*S*^) and dysfunction of the repair genes (*mei-41*, *mus101*, *mus210*, *mus304*, and *mus309*). Blue arrow – statistically significant (*p* < 0.01) transgenerational effects of irradiation in individuals with dysfunction of the repair genes and without induction of *hobo* elements; red arrow – statistically significant (*p* < 0.01) transgenerational effects of irradiation in individuals with dysfunction of the repair genes in the presence transposition of *hobo* elements; yellow arrow – stimulating effect (*p* < 0.01) of parental radiation exposure on fertility in females with dysfunction of the repair genes and in the presence of transpositions of *hobo* elements; asterisk – negative effect (*p* < 0.01) of induction of *hobo* elements on fertility of females.

The results show (Figure 3) that, as well as *HiR*^*S*^, the F_1_ offspring with disfunction of the *mei-41*, and *mus309* genes and with high *hobo* activity have low (*p* < 0.05–0.01) fertility. This tendency is observed in the F_2_ offspring with disfunction of the *mei-41* gene irrespective of parental radiation exposure. Despite the fact that the fertility of the offspring with disfunction of the *mus304* gene is higher than the fertility of the above variants, they experience the effect of parental radiation exposure (*p* < 0.01) which persists in subsequent generations. Along with the negative effects of radiation, the stimulating effect of chronic exposure to low doses of radiation on the reproductive rate was observed. This is typical of the F_1_ offspring with disfunction of the *mei-41* and *mus101* genes, the parental exposure of which led to an increase in fertility.

### Survival

**FIGURE 4:**
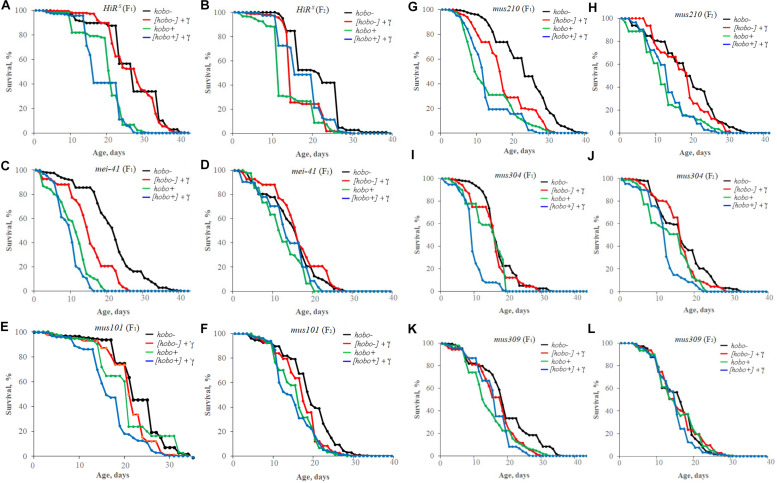
Effect of a chronic irradiation in low doses and induction of the *hobo* elements on the survival of *D. melanogaster* male imago F_1_ **(A,C,E,G,I,K)**/F_2_ **(B,D,F,H,J,L)** with normal repair genes [*HiR*^*S*^ **(A,B)**] and dysfunction of the repair genes: *mei-41* **(C,D)**, *mus101* **(E,F)**, *mus210* **(G,H)**, *mus304* **(I,J)**, and *mus309* **(K,L)**.

The influence of parental irradiation either did not have a statistically significant effect or led to a decrease in the median survival by 7.7–29.6% (*p* < 0.01–0.001) in all F_1_ offspring (except for offspring with disfunction of the *mus304* gene) and the F_2_ offspring of *HiR*^*S*^ strain without *hobo* elements (Table 3 and Figure 4). The maximum lifespan was reduced only in specimens with dysfunction of the *mei-41* (in F_1_/F_2_ offspring), *mus210* (in F_1_ offspring), *mus304* (in F_2_ offspring), and *mus309* (in F_1_ offspring) genes by 8.8–26.5% (*p* < 0.05–0.01). However, in the presence of *hobo* elements in the genomes of *D. melanogaster* strains, not only the absence of changes in the survival rate and its decrease but also an increase in this parameter were found. Parental radiation exposure had an adverse effect (*p* < 0.01–0.001) on the median lifespan of F_1_ specimens with dysfunction of the *mei-41*, *mus101*, and *mus304* genes. At the same time, a decrease in the maximum lifespan (by 17.6%, *p* < 0.05) occurred only in offspring with dysfunction of the *mus101* gene. We observed an increase in survival rate in the next generation. Thus, in F_2_ offspring *HiR*^*S*^ with normal functioning of the repair genes, the median survival rate increased by 25.6% (*p* < 0.01). A significant increase in maximum lifespan (by 5%, *p* < 0.01) was also recorded in F_2_ offspring with dysfunction of the *mei-41* gene.

**TABLE 3:** Effects of DNA repair genes and system of radiation-induced activation of *hobo* elements on the lifespan of *D. melanogaster* male imago.

Genotype	Variant	Generation	M	dM	Cox-Mantel test	90%	d90%	Wang-Allison test
*HiR*^*s*^	*hobo-*	F_1_	28.7			38		
*HiR*^*s*^	[*hobo-*] + γ	F_1_	28.1	−2.09	*p* > 0.05	36	−5.26	*p* > 0.05
*HiR*^*s*^	*hobo*+	F_1_	21.5			26		
*HiR*^*s*^	[*hobo*+] + γ	F_1_	19.9	−7.44	*p* > 0.05	25	−3.84	*p* > 0.05
*HiR*^*s*^	*hobo-*	F_2_	22.5			28		
*HiR*^*s*^	[*hobo-*] + γ	F_2_	17.7	−21.3	***p* < 0.001**	25	−10.7	*p* > 0.05
*HiR*^*s*^	*hobo*+	F_2_	15.6			25		
*HiR*^*s*^	[*hobo*+] + γ	F_2_	19.6	25.6	***p* < 0.01**	28	12.0	*p* > 0.05
*mei-41*	*hobo-*	F_1_	23.6			34		
*mei-41*	[*hobo-*] + γ	F_1_	16.6	−29.6	***p* < 0.001**	25	−26.5	***p* < 0.01**
*mei-41*	*hobo*+	F_1_	12.3			17		
*mei-41*	[*hobo*+] + γ	F_1_	11.2	−8.94	***p* < 0.01**	15	−11.7	*p* > 0.05
*mei-41*	*hobo-*	F_2_	16.7			25		
*mei-41*	[*hobo-*] + γ	F_2_	18.3	9.58	*p* > 0.05	26	4.00	*p* > 0.05
*mei-41*	*hobo*+	F_2_	14.1			20		
*mei-41*	[*hobo*+] + γ	F_2_	14.4	2.12	*p* > 0.05	21	5.00	***p* < 0.01**
*mus101*	*hobo-*	F_1_	24.8			31		
*mus101*	[*hobo-*] + γ	F_1_	22.9	−7.66	***p* < 0.01**	30	−3.33	*p* < 0.05
*mus101*	*hobo*+	F_1_	22.3			34		
*mus101*	[*hobo*+] + γ	F_1_	18.9	−15.2	***p* < 0.001**	28	−17.6	***p* < 0.05**
*mus101*	*hobo-*	F_2_	20.7			27		
*mus101*	[*hobo-*] + γ	F_2_	18.7	−9.66	***p* < 0.05**	24	−11.1	*p* > 0.05
*mus101*	*hobo*+	F_2_	17.3			23		
*mus101*	[*hobo*+] + γ	F_2_	16.7	−3.47	*p* > 0.05	24	4.34	*p* > 0.05
*mus210*	*hobo-*	F_1_	25.1			34		
*mus210*	[*hobo-*] + γ	F_1_	19.5	−22.3	***p* < 0.001**	31	−8.82	***p* < 0.01**
*mus210*	*hobo*+	F_1_	14.2			24		
*mus210*	[*hobo*+] + γ	F_1_	14.1	−0.70	*p* > 0.05	25	4.16	*p* > 0.05
*mus210*	*hobo-*	F_2_	20.9			31		
*mus210*	[*hobo-*] + γ	F_2_	19.3	−7.65	*p* > 0.05	29	−6.45	*p* > 0.05
*mus210*	*hobo*+	F_2_	13.7			25		
*mus210*	[*hobo*+] + γ	F_2_	14.8	8.03	*p* > 0.05	23	−8.00	*p* > 0.05
*mus304*	*hobo-*	F_1_	18.1			25		
*mus304*	[*hobo-*] + γ	F_1_	16.9	−6.63	*p* > 0.05	25	0.00	*p* > 0.05
*mus304*	*hobo*+	F_1_	14.7			20		
*mus304*	[*hobo*+] + γ	F_1_	11.3	−23.1	***p* < 0.001**	14	−30.0	*p* > 0.05
*mus304*	*hobo-*	F_2_	17.9			27		
*mus304*	[*hobo-*] + γ	F_2_	17.3	−3.35	*p* > 0.05	22	−18.5	***p* < 0.05**
*mus304*	*hobo*+	F_2_	14.7			21		
*mus304*	[*hobo*+] + γ	F_2_	14.7	0.00	*p* > 0.05	21	0.00	*p* > 0.05
*mus309*	*hobo-*	F_1_	21.1			32		
*mus309*	[*hobo-*] + γ	F_1_	18.1	−14.2	***p* < 0.001**	27	−15.6	***p* < 0.05**
*mus309*	*hobo*+	F_1_	15.9			25		
*mus309*	[*hobo*+] + γ	F_1_	17.0	6.92	*p* > 0.05	22	−12.0	*p* > 0.05
*mus309*	*hobo-*	F_2_	17.3			24		
*mus309*	[*hobo-*] + γ	F_2_	17.2	−0.57	*p* > 0.05	25	4.16	*p* > 0.05
*mus309*	*hobo*+	F_2_	17.1			24		
*mus309*	[*hobo*+] + γ	F_2_	16.1	−5.85	*p* > 0.05	22	−8.33	*p* > 0.05

*(*hobo*-) – control variant, without *hobo* elements; (*hobo*+) – variant with *hobo* elements; ([*hobo*-] + γ) – variant without *hobo* elements, with exposure 120.9 mGy; ([*hobo*+] + γ) – variant with *hobo* elements, with exposure 120.9 mGy; M– (days); 90% - age of 90% mortality (days); dM, d90% - the differences in median lifespan and age of 90% mortality estimated between *hobo*- and [*hobo*-] + γ, *hobo*+ and [*hobo-*] + γ. Bold values denoted that statistically significant transgenerational effects of irradiation.*

## Discussion

The increased level of transpositional activity of functional transposons in genetic dysgenic systems suggests a high sensitivity of genotypes to the action of DNA-damaging factors and disruptions in the processes of cellular defense. This is confirmed not only by numerous studies of other authors, but by our own experimental facts. Both irradiation and mutations in stress-response genes have been shown to affect the effects of TE transpositions under dysgenic conditions (Yushkova and Zainullin, 2016). This is reflected in an increase of sterility, the frequency of locus-specific mutability, recessive and dominant lethal mutations (Margulies et al., 1986, 1987; Yushkova and Zainullin, 2014). The effects of irradiation and transposon activity are associated with the induction of DSBs (Sobels and Eeken, 1981; Langen et al., 2020). As shown in previous studies, certain stress-response genes are involved in sensing these types of DNA damage. Among them, there are genes that are turned on in response to DNA damage induced by either *P*-transpositions or irradiation (Yushkova and Zainullin, 2016).

In this work, if we consider these factors separately, we can see that the manifestation of transgenerational instability in *D. melanogaster* as a result of induction of *hobo* elements is stronger than as a result of low-dose irradiation. Since in this experiment we used strains of the E-cytotype, the observed effects are mainly due to the influence of dysgenesis. Given this fact, it can be assumed that defects in the DNA repair processes can enhance those dysgenic changes that are accompanied by cell death and dominant lethality (Slatko et al., 1984). The data obtained indicate that defects in excision repair, post-replicative DNA repair and recombination (*mus304* and *mei-41* genes), as well as DSB repair (*mus304* and *mus309* genes) affect the frequencies of DNA damage and DLMs during *hobo* element transpositions. At the same time, against the background of relative sensitivity to *hobo* factors (in parameters of GD sterility, Mt and DLM), a positive effect of disfunction of the *mus101* gene was noted, which was manifested in the form of a decrease in DNA damage and an increase in offspring fertility in response to *hobo* transposition, both with and without irradiation. The *mus101* gene is known to be involved in post-replicative DNA repair and meiosis it is also involved in repair of DSBs induced by low-dose radiation in germline cells (Gatti, 1979; Yushkova, 2020). In mitotically active somatic tissues, the mechanisms associated with the *mus101* gene (control of the cell cycle, non-recombination post-replicative DNA repair) are obviously less active than the mechanisms of post-replicative and excision repair, recombination repair (*mus304* and *mei-41* genes) and DSB repair (*mus309* gene).

Note that “DLM” is one of the reliable parameters that has been used in studies of the transgenerational effect irradiation in different animal organisms (from *Drosophila* to humans) for more than 40 years (Luning et al., 1976; Zainullin et al., 1992; O’Reilly et al., 1994; O’Reilly and Mothersill, 1997; Creane et al., 1999; Mothersill et al., 2000; Shi et al., 2018; Vo et al., 2019). Chromosomal rearrangements that underlie the formation of DLMs in germ cells indicate the manifestation of genome instability (Mazurik and Mikhailov, 2001). Increased genetic instability is a main factor influencing the inheritance of transgenerational effects (Mothersill et al., 2017). In mammals, transgenerational inheritance of an increased level of DLM can be observed oftener in F_1_/F_2_ offspring of irradiated parents (Dubrova and Sarapultseva, 2020). In *D. melanogaster*, the inherited effects observed in the “DLM” parameter can be detected not only in the first generations but also 160 generations after the initial irradiation (E. Yushkova, L. Bashlykova, unpublished data). In our study, using this parameter we confirmed the transmission of DLMs to dysgenic and non-dysgenic F_1_ offspring of irradiated parents and showed that the formation of transgenerational effects of irradiation depends on the functional level of post-replicative and excision repair and DNA DSB repair. Inheritance of the increased frequency of radiation-induced DLM by subsequent generations is mainly due to the transposition activity of *hobo* elements and the level of the *mus304* gene activity which is responsible for the complex of repair systems. It is known that the increased frequency of *hobo* transpositions can persist for many tens of generations after inducing crosses (Yurchenko et al., 2011).

In the offspring of unirradiated parents, the influence of the *mei-41*, *mus304*, and *mus309* (only in F_2_ offspring) genes on the level of *hobo* transpositions was shown.

This indicates the existence of interaction between the processes of TE transpositions and the mechanisms of cell defense during transgenerational inheritance. The importance of the existence of various systems of DNA repair in the cell, in particular, post-replicative and excision repair, is confirmed by studies of the viability in the *mei*- and *mus*-strains undergoing *P*-element transpositions. It turned out that specimens with both disfunctions in the DNA repair system and active *P*-elements in the genome have high sterility, low fertility, and premature aging of male germline cells (Margulies, 1990; Yushkova and Zainullin, 2016). Analyzing our own results and literature data, we can confidently speak about the differences in the responses of mutant genotypes to the action of one or another TE. The exception is specimens with a mutation in the *mus304* gene which equally respond to the transposition of the *P* and *hobo* elements. Other strains with disfunction of the genes (*mus101* and *mus309*), despite their sensitivity to both transposons, showed conflicting effects on the levels of DNA damage, survival and fertility of the offspring. Only in terms of DLM and sterility parameters, both transposons were equally effective, which confirms the existence of a common mechanism for the formation of P-M and H-E genetic systems in early ontogenesis. In the early stages of development, transposons are highly active and cause serious genetic damage. Such genetic abnormalities lead to the death of germline cells, resulting in sterility of the offspring (Niki and Chigusa, 1986).

Sterility characterizes the changes that take place in the germline cells. In *Drosophila* females, DNA damage in germline cells causes cell cycle arrest and apoptosis, which explains the absence of developing egg chambers under the mass transpositions (Hassel et al., 2014; Shim et al., 2014). The p53 transcription factor and the Chk2 protein (Checkpoint kinase 2) are of great importance for protecting germline cells from DNA damage. Studies of their role in DNA damage repair after transpositions have shown that mutations in *Chk2* suppress transposon-induced ovarian atrophy, while mutations in *p53*, on the contrary, aggravate female sterility. The authors concluded that the *p53* gene is required to make female germline cells tolerant to TE activity (Tasnim and Kelleher, 2018). It is possible that the *mei-41* and *mus304* genes can also suppress sterility during *hobo* transpositions. On the other hand, the *mus101*, *mus210*, and *mus309* genes can aggravate the sterilizing effect in the presence of *hobo* elements. The role of *mei-41* and *mus304* genes in maintaining the integrity of germline cells under conditions of intracellular stress is obvious. To test this assumption, further studies of the effect of *mus* genes on the resistance of germline cells to transcriptionally active TEs are required.

The diverse effects observed in specimens with the induced activity of *P* and *hobo* elements are associated with the functional features of each TE, as well as with the specificity of the regulation of their transpositions. For example, the *P* element is active only in dysgenic conditions; the *hobo* transposon can be active without dysgenesis (Calvi and Gelbart, 1994; Zakharenko et al., 2006). The *P* element activity is limited only to germline cells due to regulated mRNA splicing (Laski et al., 1986). The activity of *hobo* elements is observed both in germline cells and in somatic tissues (Calvi and Gelbart, 1994; Handler and Gomez, 1995). At the same time, the *hobo* transcriptional activity in the somatic genome is low and is associated with a weak, although statistically significant, activity of the promoter of *hobo* (Blackman et al., 1987; Palazzo et al., 2019). The promoters of *hobo* element are more constructed, can contain several core-promoter motifs, and have AT and GC sequences in equal proportions. This structural organization of the promoters of *hobo* differs from the structure of highly active promoters of *Tc1/mariner* transposons that are AT-rich in sequences, have divergent or absent core-promoter motifs, and may be unevenly distributed. As a result, the promoters of *Tc1/mariner* elements are called “blurry” promoters which are capable of inducing gene transcription not only within one genome of one species, but also in distant genomes (Palazzo et al., 2019). We assume that despite a weak *hobo* promoter activity in the somatic genome, under dysgenic conditions, the *hobo* promoter activity increases. This leads to a weakening of the transcriptional regulation of transposase expression and a subsequent increase in the transposition activity of *hobo* elements.

Unlike the *P* element, the tissue-specific *hobo* transposition is regulated by the expression of transposase at the transcriptional level (Calvi and Gelbart, 1994). The frequency of transpositions of such TEs in dysgenic (inducing) crosses is regulated by the level of reactivity of females that is inherited mainly through the maternal line (Udomkit et al., 1996). In this case, the level of reactivity is closely related to the DNA repair and recombination processes and is enhanced by the action of DNA damaging factors (Bregliano et al., 1995). Such interaction is believed to be one of the manifestations of the unified inducible repair-recombination system VAMOS (Variability Modulation System) which, like the SOS response system in bacteria, is involved in modifying the level of variability in response to unfavorable environmental conditions (Laurençon et al., 1997). The molecular mechanisms of the formation of this system have not yet been clarified. To date, there have been several studies supporting the existence of this system. These are the works of Bregliano et al. (1995) and Margulies et al. (1986, 1987); Margulies (1990) describing the participation of *mei-9* and *mei-41* genes both in control of recombination repair processes and in determination of the level of reactivity (Margulies et al., 1986, 1987; Margulies, 1990; Laurençon and Bregliano, 1995). Our research was also aimed at the development of this hypothesis. The *mus* genes we are studying belong to the *mei* group, the mutations of which are known to affect meiosis. The use of chronic low-intensity irradiation (which is a factor that organisms in nature constantly encounter) and genetic systems with mutations of the *mus* genes can significantly clarify the work of molecular mechanisms of adaptation.

The reaction of offspring with disfunction in the *mei-41* and *mus* genes undergoing transposition of *hobo* elements after chronic radiation exposure depends on the studied parameter. The most pronounced effect of parental radiation exposure was observed in dysgenic offspring with disfunction of the *mus304* gene – in all parameters and in two generations (considering the conditionally positive (*mei-41*) and negative (*mus210*) controls). Dysgenic F_1_/F_2_ offspring of *mus309* strain derived from irradiated parents showed visible effects only at the level of GD sterility, DLMs and DSBs. Specimens with disfunction of the *mus101* and *mus210* genes reacted only at the level of DLMs and survival, and only in the first generation. The transgenerational effects of irradiation were observed also in the offspring of *mei-41* strain in presence of *hobo* elements, but only in the first generation. It follows from this that the studied interactions of TE transpositions and mutations in DNA repair genes have a general long-term effect on survival. Like *mei-41*, the *mus304* gene is a with a conserved function in the evolution gene capable of participating in the DNA damage repair after TE transpositions or irradiation. The effect of the *mei-41* mutation on the recombination frequency which increases as a result of transpositions of *P* elements is well known (Chmuzh et al., 2006). In this case, the recombinations frequency increases upon irradiation (Zakharenko et al., 2006). Since the *mus304* gene is responsible for recombination repair, a mutation in this gene is likely to also affect the recombination processes against the background of increased activity of *hAT*-transposase (an enzyme involved in the *hobo* transpositions). Transposase is structurally similar to the V(D)J-recombinase (recombination enzyme) which can cause DNA break during V(D)J-recombination (Zhou et al., 2004). This relationship between the systems of V(D)J-recombination and transpositional activity may significantly contribute to the frequency of DSBs, increasing their level in response to irradiation. Moreover, this type of recombination leads to extensive deletions and duplications, the accumulation of which can lead to cell death and, as a result, to the death of the organism (Preston et al., 1996). In mammals, the *ATRIP* gene (a homolog of the *mus304* gene) plays a dominant role in recombination. Like the *ATM* gene (the homolog of the *Drosophila mei-41* gene), it is involved in the DSB repair along the homologous recombination pathway (Koromyslov et al., 2003; Chmuzh et al., 2006). Since both *P* and *hobo* transpositions and irradiation cause the formation of DSBs, it is possible that the *mus304* gene has an additional function of repairing such types of DNA damage, reducing the level of transgenerational instability.

The offspring with dysfunction in the *mus309* gene turned out to be sensitive, although to a lesser extent, to transpositions and parental radiation exposure. The *mus309* gene, a human *BLM* homolog, is involved in DSB repair and in NHEJ (non-homologous end joining)-mediated DSB repair; it also has an ATP-dependent helicase activity, and is involved in NHEJ (non-homologous end joining)-mediated DSB repair (Adams et al., 2003; Portin, 2010). Recently, a new function of this gene has been identified, namely, participation in the control of the cell cycle in the organism under impact (Kondo and Perrimon, 2011). In the cell exposed to DNA damaging agents, miRNAs have been found to interact with their target genes. The level of genotoxicity strongly depends on changes in miRNA expression, in particular, dme-miR-314-3p which affects the DSB repair by targeting the *mus309* gene. Thus activated, the *mus309* gene triggers the cell cycle arrest, during which the DNA breaks are repaired (Chandraa et al., 2016).

Fertility, as one of the phenotypic traits epigenetically passed on from parents to their offspring, allows to assess the reproduction potential of the organism and reflects the number of the offspring generated over a certain period of time (Dubrova and Sarapultseva, 2020). In this work, when assessing fertility, we showed the adaptive effect of parental radiation exposure on the fertility of the F_1_ offspring with impaired function of the *mei-41* and *mus101* genes. Moreover, this property of the genes depends on the dysgenic status of the animals. The transgenerational inheritance of increased fertility by the offspring of *mei-41* (in combination with other parameters) obtained from irradiated parents in non-dysgenic conditions (without *hobo* transpositions) suggests that the *mei-41* gene manifests its activity mainly in sensing transposon-induced and non-radiation-induced events, and that the transmission of genome instability occurs solely due to *hobo* transpositions. On the other hand, disfunction of the *mus101* gene does not affect the inheritance by the offspring of genetic damage caused in the parental genome by radiation-induced *hobo* element transpositions. Our results on the diverse effects of parental radiation exposure and the inheritance of transpositional activity on the fertility of the offspring with disfunction of the *mus304* gene suggest that the mechanisms associated with the *mus304* gene under such radiation exposure conditions are able to maintain the integrity of germline cells until the formation of ovarioles. Maintaining the ovarian reserve at an optimal level is one of the possible adaptive strategies for dysgenic females developing under stressful environmental conditions (Yushkova, 2017).

## Summary

In this work, we for the first time assessed the involvement of DNA repair genes and transpositional activity of *hobo* elements in the inheritance of radiation-induced transgenerational instability. The specificity of the participation of the *mei-41* gene in this type of interaction was established; we found that it plays a predominant role in sensing transposon-induced rather than radiation-induced changes. At the same time, in dysgenic conditions the *mus304* gene which differs from genes of other DNA repair systems plays a decisive role in the transgenerational transmission of low-dose radiation effects. Taking into account the literature data and our own results regarding the *mus304* gene, no fact responsible for “transposon-specificity” was found. The combined action of the *mus309* gene and *hobo* transpositions on the transgenerational transmission of radiation-induced events had a long-term effect on all studied parameters only in the F_1_ offspring. Genes (*mus101* and *mus210*) that are not involved in the post-replicative recombination repair and DSB repair were found to be not involved in the repair of genetic material in the offspring of irradiated parents undergoing *hobo* transpositions.

## Data Availability Statement

Requests to access data analyzed in this study should be directed to ushkova@ib.komisc.ru.

## Author Contributions

The author conceived and designed the experiments, analyzed and interpreted the results, carried out the statistical analysis, prepared figures and tables as well as wrote the final manuscript.

## Conflict of Interest

The author declares that the research was conducted in the absence of any commercial or financial relationships that could be construed as a potential conflict of interest.

## Footnotes

1 http://autocomet.com/main_home.php

2 https://sbi.postech.ac.kr/oasis2
